# Expression and functional analyses of a Kinesin gene *GhKIS13A1* from cotton (*Gossypium hirsutum*) fiber

**DOI:** 10.1186/s12896-017-0373-2

**Published:** 2017-06-12

**Authors:** Yan-Jun Li, Shou-Hong Zhu, Xin-Yu Zhang, Yong-Chang Liu, Fei Xue, Lan-Jie Zhao, Jie Sun

**Affiliations:** 1 The Key Laboratory of Oasis Eco-agriculture, Agriculture College, Shihezi University, Bei 4 Road, Shihezi, 832003 Xinjiang China; 2 College of Life Sciences, Shihezi University, Bei 4 Road, Shihezi, 832003 Xinjiang China

**Keywords:** Kinesin13 subfamily, Cotton fiber, Trichome, *GhKIS13A1*

## Abstract

**Background:**

Cotton fiber, a natural fiber widely used in the textile industry, is differentiated from single cell of ovule epidermis. A large number of genes are believed to be involved in fiber formation, but so far only a few fiber genes have been isolated and functionally characterized in this developmental process. The Kinesin13 subfamily was found to play key roles during cell division and cell elongation, and was considered to be involved in the regulation of cotton fiber development.

**Results:**

The full length of coding sequence of *GhKIS13A1* was cloned using cDNA from cotton fiber for functional characterization. Expression pattern analysis showed that *GhKIS13A1* maintained a lower expression level during cotton fiber development. Biochemical assay showed that GhKIS13A1 has microtubule binding activity and basal ATPase activity that can be activated significantly by the presence of microtubules. Overexpression of *GhKIS13A1* in *Arabidopsis* reduced leaf trichomes and the percentage of three-branch trichomes, and increased two-branch and shriveled trichomes compared to wild-type. Additionally, the expression of *GhKIS13A1* in the *Arabidopsis Kinesin-13a-1* mutant rescued the defective trichome branching pattern of the mutant, making its overall trichome branching pattern back to normal.

**Conclusions:**

Our results suggested that *GhKIS13A1* is functionally compatible with *AtKinesin-13A* regarding their role in regulating the number and branching pattern of leaf trichomes. Given the developmental similarities between cotton fibers and *Arabidopsis* trichomes, it is speculated that *GhKIS13A1* may also be involved in the regulation of cotton fiber development.

## Background

Cotton is one of the most important commercial crops in the world. Cotton fiber, a natural fiber widely used in the textile industry, is differentiated from single cell of ovule epidermis. The development of cotton fiber consists of four overlapping stages: fiber initiation, cell elongation, secondary wall deposition and maturation [1]. A large number of genes are believed to be involved in fiber formation, but so far only a few fiber genes have been isolated and functionally characterized in this developmental process [2–6]. Therefore, further efforts to clone genes involved in fiber development and study their molecular mechanism governing fiber development are needed. The Kinesin family belongs to a class of motor proteins which can move along microtubule filaments by utilizing the energy released from ATP hydrolysis. Kinesins are present in all studied eukaryotic organisms and are implicated in a diverse range of cellular processes including intracellular transport, signal transduction, cell morphogenesis and mitosis [7–11]. Kinesins can be divided into 14 subfamilies from Kinesin-1 to Kinesin-14 based on the motor domain, a highly conserved domain with the ATP-binding and microtubule-binding activities [12]. The motor domain can be located at the C-terminus, N-terminus or in the middle of Kinesins. With the complete sequencing of many plant genomes, much research has been carried out on the Kinesin family in model plants. Sixty-one and forty-one Kinesin genes have been identified in the *Arabidopsis* and rice genome, respectively [13–15]. To our knowledge, only four Kinesin genes have been reported in cotton (*Gossypium hirsutum*). *GhKCBP*, *GhKCH1* and *GhKCH2* belonging to the Kinesin-14 subfamily were found to decorate microtubules and microfilaments and regulate their alignment and dynamic distribution [16–18]. *GhKinesin-13A* belonging to the Kinesin-13 subfamily was found to be located in the Golgi apparatus of cotton fiber based on immunofluorescent detection [19]. These studies suggested that Kinesin genes may play key roles in cotton fiber growth and developmental processes.

Much more attention was paid to the Kinesin-13 subfamily recently because of its roles in cell division and cell elongation. Mutation of *SRS3* encoding a member of the Kinesin-13 subfamily causes small and round seed phenotype in rice due to a decrease in cell length in the longitudinal direction [20]. T-DNA insertion in the *AtKinesin-13A* gene results in a sharp decrease of the size and number of Golgi vesicles in root cap peripheral cells [21]. Moreover, it was shown that over 70% of leaf trichomes in the *Kinesin-13a-1* mutants bore four branches, while the trichomes of wild-type (Col-0) predominantly bore three branches [19]. Both cotton fibers and *Arabidopsis* trichomes are derived from single cells of epidermis, and likely to be regulated by a similar development mechanism. Therefore, genes that influence the development of *Arabidopsis* trichome may also play key roles in cotton fiber development. In a previous study, we isolated seven putative *G. hirsutum* homologues of *AtKinesin-13A* using primers designed based on the genome sequence of *G. raimondii*, and analyzed the sequence characteristics of these Kinesin genes [22]. Of the seven *Kinesin-13* genes, *GhKIS13A1* was found to be virtually identical to the previously reported *GhKinesin-13A*, a homologue of the *Arabidopsis AtKinesin-13A* [19]. In this study, on the basis of expression pattern and biochemical characterization of *GhKIS13A1*, we showed that *GhKIS13A1* not only influences the number and branching patterns of *Arabidopsis* leaf trichomes but also is able to rescue the mutant phenotype of the *Arabidopsis Kinesin-13a-1* mutant, suggesting that *GhKIS13A1* may play an important role in cotton fiber development and is a potential candidate for improving cotton fiber quality by genetic engineering.

## Methods

### Materials

Flowers and fibers were collected from the cotton (*Gossypium hirsutum*) variety “Xinluzao 36” plants grown in a normal agronomic field. Flower buds on the anthesis day were tagged and marked as 0 day post anthesis (DPA). Bolls were collected at 3, 6, 9, 12, 15, 18, 21, 24 and 27DPA. Ovules were excised from the bolls and fibers were scraped from the ovules. The seeds of “Xinluzao 36” were sterilizated by 0.1% HgCl_2_ for 10 min, washed with sterile water 3–4 times and then sown on the Murashige and Skoog (MS) ager medium. The root, hypocotyl and leaf were collected from the tissue-cultured seedlings grown for about 2 weeks. All the collected materials were immediately frozen in liquid nitrogen and stored at -80°C until RNA extraction. The ecotype Columbia (Col-0) of *Arabidopsis thaliana* and the *Kinesin-13a-1* mutant were used in transgenic analysis.

### Cloning of *GhKIS13A1*

Total RNAs were extracted from cotton fibers and then reverse transcribed to generate cDNA. Gene-specific primers KIS-1: 5′-ATG GGT GGC CAG ATG CAG CAA AGC-3′ and KIS-2: 5′-ACG AGG AAC TCT TTT CCG ACT C-3′ were designed based on the *GhKIS13A1* sequence (accession number, KP036626) downloaded from the GenBank database. The open reading frame (ORF) of *GhKIS13A1* was amplified by PCR using the gene-specific primers and cotton fibers cDNA as a template. The PCR amplification program was: 94°C for 3 min, 30 PCR cycles (94°C for 1 min, 56°C for 1 min and 72°C for 2.5 min), and 72°C for 10 min. The PCR product was purified and cloned into the pGEM-T easy vector (Promega, USA), and then transformed into *E. coli* DH10B cells for sequencing.

### Phylogenetic analysis

Kinesin proteins from *Arabidopsis*, rice, and corn were obtained from the GenBank database (http://www.ncbi.nlm.nih.gov/genbank/). Thirteen cotton Kinesins were obtained from the genome database of *Gossypium hirsutum* (www.cottongen.org/) by using GhKIS13A1 as a probe. MEGA 4.0 was used for the construction of phylogenetic tree, using the neighbor-joining method and the bootstrap test carried out with 1000 replicates.

### Expression pattern of *GhKIS13A1* in different cotton organs

Total RNAs from cotton roots, hypocotyls, leaves, flowers, ovules and fibers were extracted according to a previous method [23], and reversely transcribed into cDNAs. Then, the cDNAs were used as templates in Real-time Quantitative PCR (qPCR) reactions with gene-specific primers. The forward primer KIS-5 is 5′-CTG GTC GAA GGG TAG CAG AG-3′, and the reverse primer KIS-6 is 5′-GGC TCG AAG AAC CAC CAT AA-3′. The cotton *UBI* gene was used as an internal reference for normalization of cDNA templates. The forward primer of *UBI* is 5′-CAG ATC TTC GTA AAA CCC T-3′, and the reverse primer is 5′-GAC TCC TTC TGG ATG TTG TA-3′. The qPCR reactions were conducted using a SYBR Green I Master mixture (Roche, Switzerland) on a LightCycler 480IIsystem (Roche, Switzerland) under conditions of an initial denaturation at 95 °C for 2 min followed by 40 cycles of denaturing at 95°C for 15 s, annealing at 55 °C for 20 s, and extending at 72 °C for 15 s. The qPCR reaction was repeated three times. Quantification was performed using the 2^-△△Ct^ method.

### Expression and purification of the recombinant GhKIS13A1 protein

The *GhKIS13A1* ORF was cloned into plasmid pET28a (+) (with a His tag) using *Eco*R1/*Hin*dIII restriction sites to generate the pET28a-*GhKIS13A1* construct. After sequencing confirmation, the pET28a-*GhKIS13A1* construct was transformed into *E. coli* BL21 (DE3) cells for fusion protein expression. A single colony of *E.coli* BL21 cells harboring the recombinant plasmid pET28a-*GhKIS13A1* was incubated at 37 °C until the OD_600_ reached about 0.6, and protein synthesis was induced by adding 0.8 mM isopropyl thio-β-D-galactoside (IPTG) for up to 6 h at 37°C. Protein purification was performed with His-Binding-Resin following the manufacturer’s instructions (Yuekebio, Shanghai, China). The purity of the GhKIS13A1-His fusion protein was loaded on 12% SDS-PAGE gel for western blot experiment with anti-6 × His antibody (Sigma). The protein concentration was determined by the Bradford method with bovine serum albumin (BSA) as the standard.

### Assays for Kinesin activity of GhKIS13A1

Microtubule binding assay was performed according to a previous method [24]. To prepare microtubules, the tubulin protein (GenMed Scientifics Inc. USA) was polymerized by incubating with 2 mmol•L^−1^ GTP and 30 μmol•L^−1^ paclitaxel for 30 min at 37 °C. A volume of 50 μl microtubules (2.7 mg•mL^−1^) was mixed with 50 μl purified GhKIS13A1 (0.44 mg•mL^−1^) in the absence or presence of 10 mmol•L^−1^ ATP and 5 mmol•L^−1^ 5’-adenylylimidodiphosphate (AMP-PNP; a non-hydrolyzable ATP analogue). After incubation for 15 min at 37 °C, the reactions were centrifuged at 14,000 g for 30 min. The supernatant and pellet were electrophoresed by SDS-PAGE and stained with Coomassie Brilliant Blue R-250.

To analyze the ATPase activity and microtubule-activated ATPase activity, 100 μl purified GhKIS13A1 protein (0.44 mg•mL^−1^) was incubated with 100 μl microtubulins (2.7 mg•mL^−1^) for 15 min at 37°C. Then 20 μl ATP (20 mmol•L^−1^) was added and incubated for another 15 min. The reaction was terminated by adding 20 μl of 100% trichloroacetic acid followed by placing on ice for 10 min. After 10 min of centrifugation at 4°C, the supernatant was collected and used to determinate the inorganic phosphate according to the LeBel’s method [25].

### Vector construction and *Arabidopsis* transformation

The *GhKIS13A1* ORF was amplified using KIS-1 and KIS-2 primers and then constructed into plant expression vector pGWB17 (with a 35S promoter) by the Gateway technology. To ensure the sequence conformity to the entry vector in the right direction in the process of vector construction, the recognition sequence ‘CACC’ was added to the 5’ end of the forward primer KIS-1. The PCR product was integrated into the entry vector pENTR/D-TOPO following the instructions of pENTR™ Directional TOPO® Cloning Kits (Invitrogen, USA). After confirmed by DNA sequencing, the entry vector carrying the *GhKIS13A1* ORF was mixed with expression vector pGWB17 for LR recombination reaction following the instructions of Gateway® LR Clonase™ II Enzyme Mix (Invitrogen, USA). The confirmed plasmid was transformed into *Agrobacterium tumefaciens* GV3101 by the standard transformation method [26]. *Arabidopsis* ecotype Col-0 and the *Kinesin-13a-1* mutant were used in transformation by the floral dip method [27] to generate transgenic lines overexpressing *GhKIS13A1* (*GhKIS13A1*-OX) and complementing the *Kinesin-13a-1* mutation (*GhKIS13A1*-COM), respectively.

### PCR and qPCR detection of *Arabidopsis* transgenic plants

Genomic DNA was extracted from *Arabidopsis* leaves and used in PCR analysis for detection of the presence of the inserted *GhKIS13A1* sequence. The gene-specific primers KIS-1 and KIS-2 were used to detect exogenous *GhKIS13A1* transformations. PCR-positive plants harboring the *GhKIS13A1* gene were further analyzed for its expression level by qPCR. Total RNA was extracted from 9-day-old wild-type, transgenic lines and the *Kinesin-13a-1* mutant using TRIZOL Reagent (Tiangen, China), and reversely transcribed into cDNAs. Then, the cDNAs were used as templates in qPCR reactions with gene-specific primers KIS-5 and KIS-6. The *Arabidopsis ACTIN2* gene was used as an internal reference for normalization of cDNA templates. The forward primer of *ACTIN2* is 5′-GGT AAC ATT GTG CTC AGT GGT GG, and the reverse primer is 5′-AAC GAC CTT AAT CTT CAT GCT GC-3′.

### Analysis of leaf trichomes of *Arabidopsis* transgenic plants

Six *GhKIS13A1*-OX and *GhKIS13A1*-COM transgenic lines, each with seven randomly selected plants, were used in leaf trichome analysis. The leaf of the same part on each plant was picked for counting the total number of trichomes and calculating the percentages of trichomes with different number of branches.

## Results

### Analysis of phylogenetic relationships

**Fig. 1:**
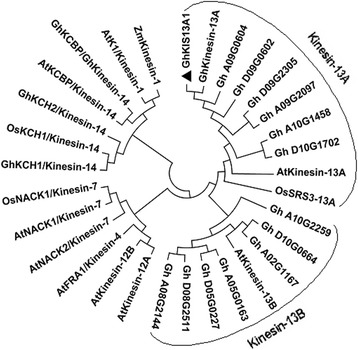
Phylogenic relationship of GhKIS13A1 and other Kinesin proteins. The phylogenetic tree was constructed by MEGA 4.0 using the neighbor-joining method and the bootstrap test carried out with 1000 bootstrap replicates. The sequence used are GhKIS13A1 (*Gossypium hirsutum*, KP036626), GhKinesin-13A (*Gossypium hirsutum*, AAQ18797), AtKCBP/Kinesin-14 (*Arabidopsis thaliana*, AAC37475), GhKCBP/GhKinesin-14 (*Gossypium hirsutum*, AAP41107), AtKinesin-13A (*Arabidopsis thaliana*, AAL07208), AtKinesin-13B (*Arabidopsis thaliana*, AAK96543), AtKinesin-12A (*Arabidopsis thaliana*, AAF78893), AtKinesin-12B (*Arabidopsis thaliana*, AEE76799), AtK1/Kinesin-1 (*Arabidopsis thaliana*, BAC03248), OsNACK1/Kinesin-7 (*Oryza sativa*, BAB86283), GhKCH1/Kinesin-14 (*Gossypium hirsutum*, AAW03152), GhKCH2/Kinesin-14 (*Gossypium hirsutum*, ABO28522), OsSRS-13A (*Oryza sativa*, EEE62380), ZmKinesin-1 (*Zea mays*, DAA35856), OsKCH1/Kinesin-14 (*Oryza sativa*, NP_001066967), AtFRA1/Kinesin-4 (*Arabidopsis thaliana*, AAN86114), and 13 Kinesins from cotton genome database by using GhKIS13A1 as a probe, including Gh_D09G0602, Gh_A09G0604, Gh_A09G2097, Gh_D09G2305, Gh_D10G1702, Gh_A10G1458, Gh_A10G2259, Gh_D10G0664, Gh_A05G0163, Gh_D05G0227, Gh_D08G2511, Gh_A08G2144, Gh_A02G1167

In the genome database of *Gossypium hirsutum*, thirteen Kinesin13 proteins were identified by using the protein sequence of GhKIS13A1 as a probe. Along with sequences from *Arabidopsis*, rice and corn, a phylogenetic tree was constructed (Fig. 1). These proteins from other plants are members of either the Kinesin-13 subfamily or the other subfamilies, including Kinesin-1, Kinesin-2, Kinesin-7, Kinesin-12 and Kinesin-14 subfamilies. Phylogenetic analysis showed that all cotton Kinesins were clustered to Kinesin-13 subfamily, and divided into Kinesin-13A and Kinesin-13B, which were close to AtKinesin-13A and AtKinesin-13B, respectively (Fig. 1). GhKIS13A1 was close to GhKinesin-13A and GhA09G0604 in Kinesin-13A subclade. BLAST searches revealed that the GhKIS13A1 showed 99.56% identity with previously reported GhKinesin-13A [19], and 99.45% identity with GhA09G0604 identified from cotton database. These suggest that GhKIS13A1 was virtually identical to GhKinesin-13A and GhA09G0604, located on the A09 chromosome. The sequence differences among GhKIS13A1, GhKinesin-13A and GhA09G0604 may be caused by the difference of cotton varieties and the unavoidable small errors during sequencing or sequence assembly.

### Expression analysis of *GhKIS13A1*

**Fig. 2:**
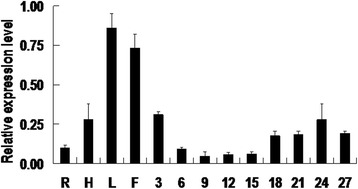
Expression pattern of *GhKIS13A1* in different cotton organs. All expression levels are relative to the cotton *UBI* gene that was used as an internal control. R: root; H: hypocotyl; L: leaf; F: flower; 3: 3DPA ovule; 6-27: the fiber cells at 6, 9, 12, 15, 18, 21, 24 and 27 DPA, respectively

The qPCR was performed to investigate the expression pattern of *GhKIS13A1* in different cotton organs. The result showed that *GhKIS13A1* was expressed preferentially in flower and leaf, and at a lower level in hypocotyl, root and fiber. In contrast, *GhKIS13A1* was expressed at the lowest level in 6-15 DPA fiber cells. Although the expression of *GhKIS13A1* was observed at a slightly higher level in 3 DPA ovule and 18-27DPA fiber cells, the level were significantly lower than in the flower and leaf. In addition to 24DPA fiber cell, the expression level of *GhKIS13A1* in fiber cells was lower than in the hypocotyl (Fig. 2).

### Biochemical characterization of the motor domain in GhKIS13A1

**Fig. 3:**
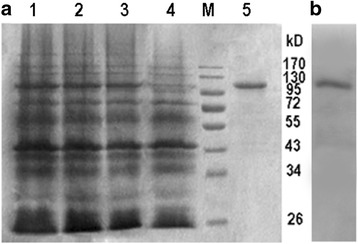
SDS-PAGE analysis of the induction and purification of GhKIS13A1 expressed in *E.coli* BL21. **a**, SDS-PAGE analysis of induction and purification of GhKIS13A1-His fusion protein. M: protein molecular marker; Lane 1: total proteins of cells after 2 h of IPTG induction; lane 2: total proteins of cells after 4 h of IPTG induction; lane 3: total proteins of cells after 6 h of IPTG induction; lane 4: total proteins of cells without IPTG induction; lane 5: purified GhKIS13A1-His fusion protein. **b**, GhKIS13A1-His fusion protein confirmed by western blot experiment with anti-6 × His antibody

To carry out the biochemical characterization of GhKIS13A1, the *GhKIS13A1* ORF was amplified and ligated into plasmid pET-28a (+) and expressed in *E.coli* BL21 cells. The GhKIS13A1-His fusion protein prepared using *E.coli* was tested in vitro for its ATPase activity and microtubulin binding activity. SDS-PAGE showed the IPTG induced production of a 110 kDa protein (Fig. 3a), consistent with the predicted molecular mass of GhKIS13A1-His. The recombinant enzyme GhKIS13A1-His was purified by His-Binding-Resin purification column. Western blot experiment showed that GhKIS13A1-His protein could be recognized by his-antibody (Fig. 3b).

**Fig. 4:**
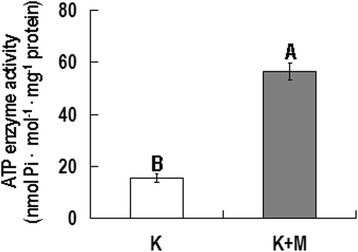
ATPase activity assays of GhKIS13A1. K: ATPase assays of GhKIS13A1 in the absence of MTs; K + M: ATPase assays of GhKIS13A1 in the presence of MTs. Data were analyzed by Paired-samples *T* test. Different uppercase letters indicate a significant difference between K and K + M at the 1% probability level

To define the ATPase activity and microtubule-activated ATPase activity, the purified GhKIS13A1-His protein was incubated with ATP in the absence or presence of polymerized microtubulins (MTs). As shown in Fig. 4, the ATPase activity of GhKIS13A1 was 15.42 ± 1.56 nmol•Pi•Min^−1^•mg^−1^ in the absence of MTs, and increased by approximately 3-fold to 56.48 ± 3.15 nmol•Pi•Min^−1^•mg^−1^ in the presence of MTs. No ATPase activity was detected in the reaction system containing MTs and lacking GhKIS13A1-His. The result suggested that GhKIS13A1 has a basal ATPase activity that can be activated significantly by the presence of MTs.

**Fig. 5:**
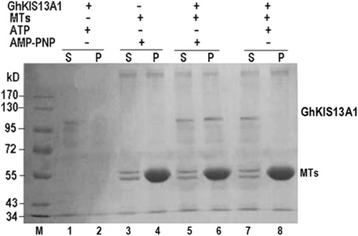
Microtubule binding assay of GhKIS13A1. M: protein molecular marker; S: supernatant; P: precipitation; lanes 1-2: microtubule binding assays of GhKIS13A1 in the absence of MTs (control test); lanes 3-4: microtubule binding assays of MTs in the absence of GhKIS13A1 (control test); lanes 5-6: microtubule binding assays of GhKIS13A1 in the presence of AMP-PNP; lanes 7-8: microtubule binding assays of GhKIS13A1 in the presence of ATP

To determine the microtubulin binding activity of GhKIS13A1, the purified GhKIS13A1-His protein was incubated with MTs in the absence or presence of either ATP or AMP-PNP, a non-hydrolyzable ATP analogue. In the absence of MTs, the GhKIS13A1-His protein remained in the supernatant after 30 min of centrifugation (Lanes 1-2 of Fig. 5). In the presence of MTs, the GhKIS13A1-His protein was co-precipitated with MTs only in the presence of AMP-PNP but not in the absence of AMP-PNP (comparing Lanes 5-6 and Lanes 7-8 of Fig. 5). These results suggest that AMP-PNP can promote the binding between GhKIS13A1 and MTs, and that GhKIS13A1 has the nucleotide-dependent microtubulin binding ability.

### Screening for *GhKIS13A1* transgenic *Arabidopsis* plants

**Fig. 6:**
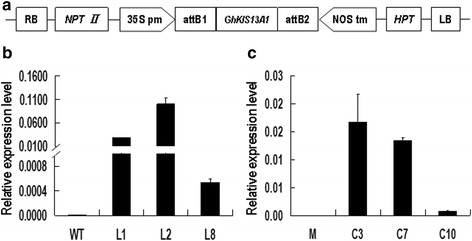
The PCR and qPCR detection of transgenic plants. **a**, Schematic representation of the expression cassettes in the plant expression vector pGWB17-*GhKIS13A1* used in *Arabidopsis* transformation. 35S pm, CaMV 35S promoter; NOS tm, NOS terminor; *HPT*, hygromycin phosphotralsferase gene; *NPT II*, neomycin phosphotransferase IIgene; attB1 and attB2, recombination site sequences in the Gateway system; *GhKIS13A1*, cDNA sequence of the *GhKIS13A1* gene; RB, right border; LB, left border; (**b**), The expression level of *GhKIS13A1* in wild-type (Col-0) and three *GhKIS13A1*-OX lines (L1, L2 and L8). WT, wild-type; (**c**), The expression level of *GhKIS13A1* in the Kinesin-13a-1 mutant and three *GhKIS13A1*-COM lines (C3, C7 and C10). M, Kinesin-13a-1 mutant. All expression levels are relative to the *Arabidopsis ACTIN2* gene that was used as an internal control

The *GhKIS13A1* overexpression vector was constructed and introduced into the *Arabidopsis* wild-type (Col-0) and the *Kinesin-13a-1* mutant plants to generate *GhKIS13A1*-OX and *GhKIS13A1*-COM lines, respectively. Successful transgenic plants were selected using PCR based on the presence of the exogenous *GhKIS13A1* gene. The T2 generation plants of three *GhKIS13A1*-OX lines (L1, L2 and L8) and three *GhKIS13A1*-COM lines (C3, C7 and C10) were selected for leaf trichome investigation. The expression levels of *GhKIS13A1* in these independent transgenic lines were quantified using qPCR. As expected, no expression of *GhKIS13A1* was detected in the wild-type (Col-0) and the *Kinesin-13a-1* mutant, while expression of *GhKIS13A1* was detected in the three *GhKIS13A1*-OX lines (L1, L2 and L3) and three *GhKIS13A1*-COM lines (C3, C7 and C10) (Fig. 6). Among the three *GhKIS13A1*-OX lines, L2 showed the highest transcript level of *GhKIS13A1*, followed by the L1 and L8 lines (Fig. 6b). Among the three *GhKIS13A1*-COM lines, C3 showed the highest transcript level of *GhKIS13A1*, followed by the C7 and C10 lines (Fig. 6c). Exogenous expression of *GhKIS13A1* in *Arabidopsis* had no effect on the overall growth and development of the transgenic plants.

### Overexpression of *GhKIS13A1* influences the number and branching of the *Arabidopsis* leaf trichomes

A previous study showed that *AtKinesin-13A* plays a role in trichome morphogenesis, and that its *Kinesin-13a-1* mutant has more trichomes with four branches than wild-type [19]. Therefore, we compared the number and branching pattern of leaf trichomes between the wild-type and the transgenic lines. The eighth rosette leaves taken from seven plants of each transgenic line were used in the comparison.

**Fig. 7:**
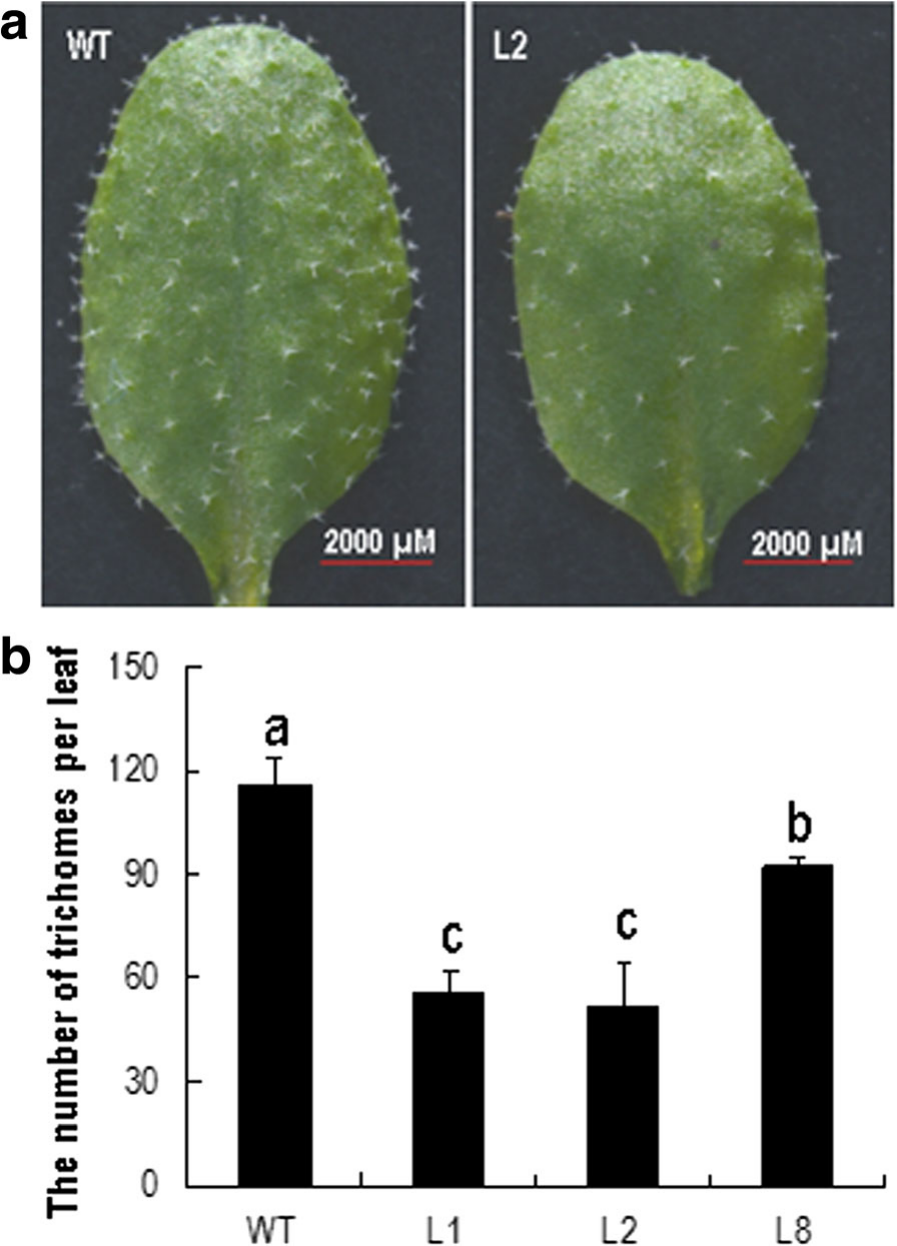
The morphology and number of leaf trichomes in the wild-type (Col-0) and *GhKIS13A1*-OX lines. **a**, Representative leaf images from the wild-type and the L2 line showing the number and distribution of trichomes. **b**, The number of leaf trichomes in the wild-type and three *GhKIS13A1*-OX lines. Different lower-case letters indicate significant differences among different lines at the 5% probability level (One-way ANOVA, Least Significant Difference test)

The number of trichomes of the three *GhKIS13A1*-OX lines were significantly less than that of the wild-type (Fig. 7), and was negatively correlated with the expression level of *GhKIS13A1* in the corresponding *GhKIS13A1*-OX line. Line L2, with the highest *GhKIS13A1* expression level (Fig. 6), produced 52 trichomes per leaf, a 54.9% reduction compared with the wild-type (116 trichomes per leaf); line L8, with the lowest expression level of *GhKIS13A1*, produced 92 trichomes per leaf, which is 20.4% less than that of the wild-type. Regarding the branching pattern, the trichomes of the wild-type predominantly had three branches (94.7%), although trichomes with four branches were occasionally seen (4.2%). In the *GhKIS13A1*-OX lines, although the majority of trichomes also had three branches, their percentages were significantly lower than that of the wild-type (Table 1). Notably, the transgenic lines produced more two-branch trichomes and shriveled-branch trichomes, suggesting that ectopic expression of *GhKIS13A1* in *Arabidopsis* has a negative effect on branching of trichomes with more than two branches. These data collectively indicated that *GhKIS13A1* overexpression can influence the number and branching of the leaf trichomes in *Arabidopsis*.

**Table 1:** Percentages of leaf trichomes with different number of branches in the wild-type and the three *GhKIS13A1*-OX lines

	No. of trichome branches			
	5	4	3	2
WT	0	4.2 a	94.7 a	0.8 c
L1	0	2.7 c	85.4 c	11.3 a
L2	0	2.0 c	86.1 c	12.0 a
L8	0	2.7 b	93.3 b	3.4 b

Data represent the mean percentages of trichomes with the indicated number of branches on the eighth rosette leaf from seven plants. Data were analyzed by one-way ANOVA. Different lower-case letters indicate significant differences among different lines at the 5% probability level (One-way ANOVA, Least Significant Difference test)

### *GhKIS13A1* is able to rescue the defective trichome phenotype of the *Kinesin-13a-1*mutant

**Fig. 8:**
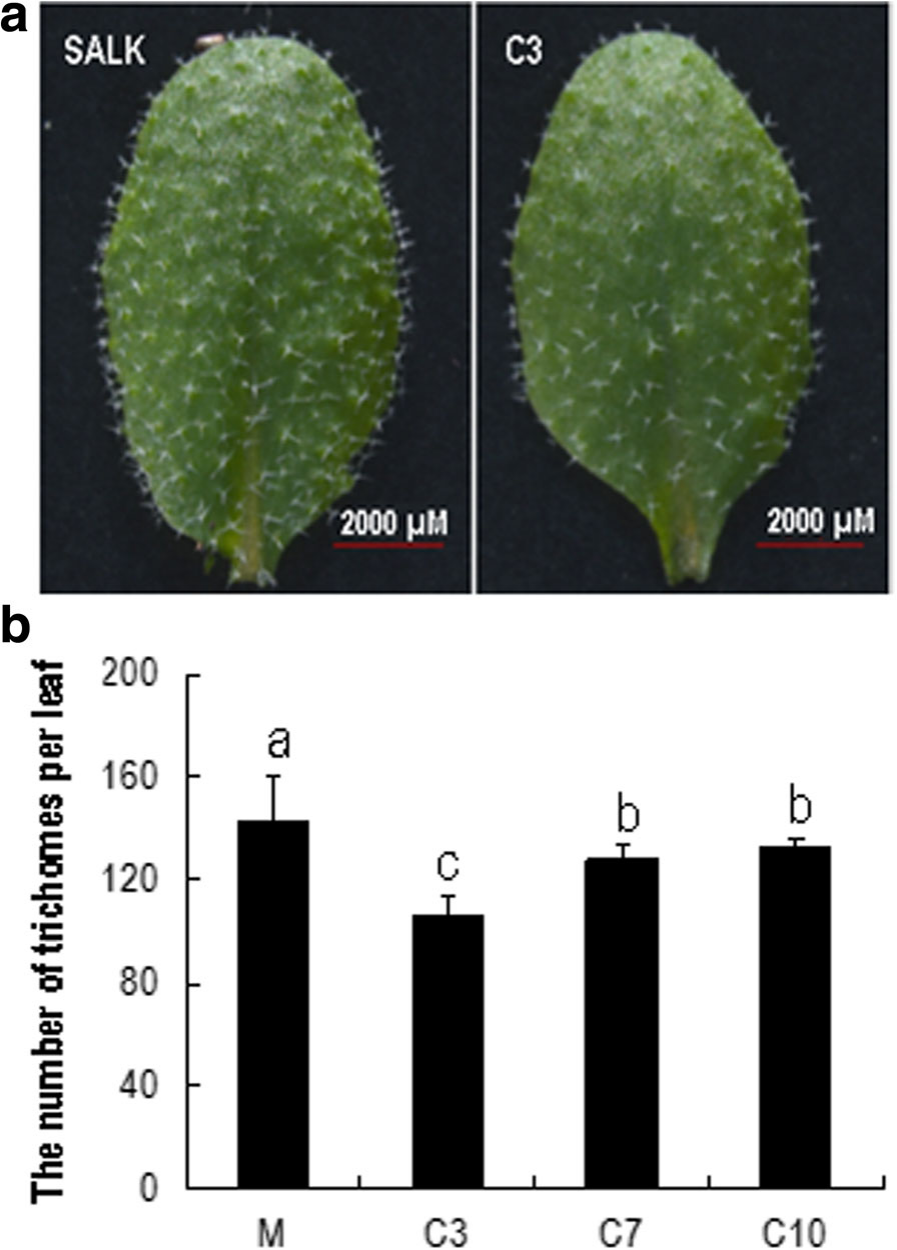
The morphology and number of leaf trichomes in the *Kinesin-13a-1* mutant and three *GhKIS13A1*-COM lines. **a**, Representative leaf images of the *Kinesin-13a-1* mutant and the C3 line showing the number and distribution of trichomes. **b**, The number of leaf trichomes in the wild-type and three *GhKIS13A1*-COM lines. Different lower-case letters indicate significant differences among different lines at the 5% probability level (One-way ANOVA, Least Significant Difference test)

To further confirm the function of *GhKIS13A1*, we carried out genetic complementation experiment by transforming the homozygous *Kinesin-13a-1* mutant with *GhKIS13A1* driven by the 35S promoter. In the three *GhKIS13A1-*COM lines (C3, C7 and C10), the number of trichomes was significantly reduced compared with the mutant plants (Fig. 8), and was negatively correlated with their corresponding *GhKIS13A1* expression levels, consistent with the results observed in the *GhKIS13A1*-OX lines. Additionally, unlike the *Kinesin-13a-1* mutant, which had most of its trichomes with four branches, the *GhKIS13A1*-COM lines had majority of their trichomes with three branches, and the percentages of such trichomes (93.0–94.9%) were similar to that of the wild-type (94.7%). As a result, the *GhKIS13A1*-COM lines had an overall branching pattern of trichomes similar to the wild-type (Table 2). These results suggest that *GhKIS13A1* is able to rescue the trichome defects of the *Kinesin-13a-1* mutant. Overall, the data observed in the transgenic plants indicated that *GhKIS13A1* and *AtKinesin-13A* has a similar function in regulating trichome development in *Arabidopsis*, but overexpression of *GhKIS13A1* in *Arabidopsis* has a negative effect on the total number of trichomes.

**Table 2:** Percentages of leaf trichomes with different number of branches in the *Arabidopsis Kinesin-13a-1* mutant and the three *GhKIS13A1*-COM lines

	No. of trichome branches			
	5	4	3	2
Mutant	1.4 a	89.3 a	8.7 b	0.7 a
C3	0 b	3.4 b	94.9 a	1.7 a
C7	0 b	4.0 b	94.4 a	1.7 a
C10	0 b	6.4 b	93.0 a	0.9 a

Data represent the mean percentages of trichomes with the indicated number of branches on the eighth rosette leaf from seven plants. Data were analyzed by one-way ANOVA. Different lower-case letters indicate significant differences among different lines at the 5% probability level (One-way ANOVA, Least Significant Difference test)

## Discussion

Kinesins are involved in a diverse number of cellular functions. It is expected that Kinesins found in cotton fiber have important cellular roles in cotton fiber development. Four cotton Kinesin genes previously reported to be associated directly or indirectly with cytoskeletons were thought to be related to fiber development [16–19]. We previously cloned 7 cDNAs encoding Kinesins in *G. hirsutum* [22]. Among them, *GhKIS13A1* was chosen for functional analysis. *GhKIS13A1* is a central motor domain Kinesin and its full length cDNA is 2730 bp encoding 909aa. Our results showed that GhKIS13A1 has microtubule-activated ATPase activity and microtubules binding activity similar to conventional Kinesins. GhKIS13A1 exhibited a relatively low basal ATPase activity under the condition of without MTs, and its ATPase activity was enhanced by approximately 3-fold in the presence of MTs. However, the 3-fold was much lower than other prokaryotic-expressed plant Kinesins and many animal Kinesins [18, 28–30]. The ATPase activity of GhKCH2 was found to be enhanced by 10-fold in the presence of MTs [18]. The Kinesins must be firstly attached to microtubules, ATP is then hydrolyzed, phosphate is released, and the Kinesins greatly reduced affinity for microtubules [29]. Therefore, much research found that AMP-PNP, the non-hydrolyzable ATP analogue, could enhance the binding of Kinesins to microtubules [30, 31]. GhKIS13A1 cannot be co-precipitated with microtubules in the presence of ATP, and can be co-precipitated with microtubules in the presence of AMP-PNP. However, some Kinesins can also co-precipitate with microtubules in the presence of ATP or in a nucleotide-free state [18, 32]. To our knowledge, this is the first report describing the biochemical characterization of a cotton Kinesin.

A number of genes with a role in the regulation of trichome number and branches have been isolated in *Arabidopsis*. Loss-of-function mutants of some of these genes showed glabrous leaves or a decease number of leaf trichomes [33–35], but for some genes, their loss-of-function mutants showed an increased clustering of trichomes [36, 37]. In addition, disruption of some genes caused trichomes with abnormal number of branches [38–40]. In this study, we showed that *Arabidopsis* plants overexpressing *GhKIS13A1* exhibited less number of trichomes than the wild-type. We also showed that the *Kinesin-13a-1* mutant harboring *GhKIS13A1* also had less number of trichomes but had the majority of its trichomes with a restored three-branch morphology. These data suggest that the expression of *GhKIS13A1* can inhibit the tichome growth of *Arabidopsis*, and can be a potential candidate for synchronously regulating the number and branching pattern in plants.

Cotton fibers are single-celled, non-glandular hairs of epidermal. Fiber initiation and fiber elongation are fairly synchronous on each ovule and progresses for 20-30 days [1]. Expression pattern analysis showed that *GhKIS13A1* was expressed at a lower level in cotton fiber (containing only trichomes), and maintained a relatively lower level during fiber initiation and elongation stages (Fig. 2). The result suggested that the lower expression of *GhKIS13A1* may be beneficial to the fiber initiation and elongation. The hypothesis is in line with the results that the lower expression level of *GhKIS13A1* produced more trichomes per leaf in six transgenic lines of *Arabidopsis*. It is speculated that *GhKIS13A1* may negatively regulate the cotton fiber development.

Both *Arabidopsis* trichomes and cotton fibers are single-celled, non-glandular hairs of epidermal. A number of studies have suggested that there are developmental similarities between cotton fibers and *Arabidopsis* trichomes [41]. *Arabidopsis* has been served as a useful model plant for dissecting the mechanisms controlling cotton fiber development. Many *Arabidopsis* homologous genes have been isolated from cotton, and some of them have been shown to play a similar role in trichome formation in *Arabidopsis* [42–44]. In this study, the expression pattern analysis and the investigation of the trichome phenotype of *Arabidopsis* harboring *GhKIS13A1* may shed insight into the possible roles of *GhKIS13A1* in cotton fiber development. The *GhKinesin13A1,* whose sequence is identical to the *GhKIS13A1*, is localized to Golgi stacks, which are frequently associated with microtubules and actin microfilaments [19]. *GhKIS13A1* may thus play a similar regulatory role in trichome morphogenesis through its effects on the organization of Golgi stacks. This paper firstly functionally characterized a Kinesin gene from cotton that may be pivotal in fiber development.

## Conclusions

In this study, biochemical characterization showed that GhKIS13A1 has microtubule binding activity, basal ATPase activity and microtubule-activated ATPase activity. *Arabidopsis* overexpressing *GhKIS13A1* exhibited less trichomes than the wild-type, and the *Kinesin-13a-1* mutant harboring *GhKIS13A1* showed less trichomes and had majority of trichomes with restored three-branch morphology, and the percentage of such trichome was matched with that in wild-type. These data suggest that *GhKIS13A1* is involved in the trichome development by regulating the total trichome number and branching. It is proposed that *GhKIS13A1* may also involve in the regulation of cotton fiber development.

## Abbreviations

AMP-PNP: 5’-adenylylimidodiphosphate
ATP: Adenosine triphosphate
BSA: Bovine serum albumin
DPA: Day post anthesis
GTP: Guanosine triphosphate
IPTG: Isopropyl thio-β-D-galactoside
MS: Murashige and Skoog
MTs: Microtubules
ORF: Open reading frame
Pi: Inorganic phosphate
qPCR: Real-time quantative PCR
SDS-PAGE: Sodium dodecyl sulfate-polyacrylamide gel electrophoresis
